# Recognition of social health: A conceptual framework in the context of dementia research

**DOI:** 10.3389/fpsyt.2022.1052009

**Published:** 2022-12-15

**Authors:** Myrra Vernooij-Dassen, Eline Verspoor, Suraj Samtani, Perminder S. Sachdev, M. Arfan Ikram, Meike W. Vernooij, Claudia Hubers, Rabih Chattat, Marta Lenart-Bugla, Joanna Rymaszewska, Dorota Szczesniak, Henry Brodaty, Anna-Karin Welmer, Jane Maddock, Isabelle F. van der Velpen, Henrik Wiegelmann, Anna Marseglia, Marcus Richards, Rene Melis, Marjolein de Vugt, Esme Moniz-Cook, Yun-Hee Jeon, Marieke Perry, Karin Wolf-Ostermann

**Affiliations:** ^1^Scientific Center for Quality of Healthcare, Radboud University Medical Center, Nijmegen, Netherlands; ^2^Department Geriatric Medicine, Radboud University Medical Center, Nijmegen, Netherlands; ^3^Discipline of Psychiatry and Mental Health, Centre for Healthy Brain Ageing (CHeBA), University of New South Wales, Sydney, NSW, Australia; ^4^Department of Epidemiology, Erasmus University Medical Center, Rotterdam, Netherlands; ^5^Harvard T.H. Chan School of Public Health, Boston, MA, United States; ^6^Department of Radiology and Nuclear Medicine, Erasmus University Medical Center, Rotterdam, Netherlands; ^7^Department of Psychology, University of Bologna, Bologna, Italy; ^8^Department of Psychiatry, Wrocław Medical University, Wrocław, Poland; ^9^Dementia Centre for Research Collaboration, Sydney, NSW, Australia; ^10^Department of Neurobiology Care Sciences and Society, Aging Research Center & Division of Physiotherapy, Karolinska Institutet, Stockholm, Sweden; ^11^MRC Unit for Lifelong Health and Ageing at UCL, Faculty of Population Health, University College London, London, United Kingdom; ^12^Department of Nursing Science Research, Institute of Public Health and Nursing Research, University of Bremen, Bremen, Germany; ^13^Department of Neurobiology, Care Sciences and Society, Division of Clinical Geriatrics, Center for Alzheimer Research, Karolinska Institutet, Stockholm, Sweden; ^14^Department of Psychiatry and Neuropsychology, School for Mental Health and Neurosciences, Faculty of Health, Medicine and Life Sciences, Alzheimer Centrum Limburg, Maastricht University, Maastricht, Netherland; ^15^Department of Clinical Psychology, University of Hull, Hull, United Kingdom; ^16^Susan Wakil School of Nursing and Midwifery, Faculty of Medicine and Health, The University of Sydney, Sydney, NSW, Australia

**Keywords:** social health, conceptual framework, dementia prevention, epidemiology, cognitive reserve, concept advancement

## Abstract

**Objective:**

The recognition of dementia as a multifactorial disorder encourages the exploration of new pathways to understand its origins. Social health might play a role in cognitive decline and dementia, but conceptual clarity is lacking and this hinders investigation of associations and mechanisms. The objective is to develop a conceptual framework for social health to advance conceptual clarity in future studies.

**Process:**

We use the following steps: underpinning for concept advancement, concept advancement by the development of a conceptual model, and exploration of its potential feasibility. An iterative consensus-based process was used within the international multidisciplinary SHARED project.

**Conceptual framework:**

Underpinning of the concept drew from a synthesis of theoretical, conceptual and epidemiological work, and resulted in a definition of social health as wellbeing that relies on capacities both of the individual and the social environment. Consequently, domains in the conceptual framework are on both the individual (e.g., social participation) and the social environmental levels (e.g., social network). We hypothesize that social health acts as a driver for use of cognitive reserve which can then slow cognitive impairment or maintain cognitive functioning. The feasibility of the conceptual framework is demonstrated in its practical use in identifying and structuring of social health markers within the SHARED project.

**Discussion:**

The conceptual framework provides guidance for future research and facilitates identification of modifiable risk and protective factors, which may in turn shape new avenues for preventive interventions. We highlight the paradigm of social health in dementia as a priority for dementia research.

## Introduction

Dementia is an urgent problem affecting 57.4 million people in 2019 worldwide (1). Most of the dementias are associated with neurodegenerative processes for which mechanisms are unclear. There is currently no cure for dementia. Our current understanding of dementia risks is primarily focused on the most common cause, namely Alzheimer’s Disease, and driven by biological factors such as the amyloid cascade hypothesis for Alzheimer’s Disease (2). While medications to manage brain amyloid exist, few effects on sustained clinical outcomes have been reported (3). Also the acknowledgment that dementia is not “normal aging” nor is it an acceleration of aging (4), suggests that these hypotheses do not tell the complete developmental story. Moreover, new findings note that age-specific incidence rates are declining in North America and Europe (5). The exploration of new pathways and hypotheses is encouraged by the recognition of neurodegenerative dementias, including Alzheimer’s disease, as multifactorial disorders that may be determined by the interplay of genetic susceptibility and environmental factors across the life course (6).

We aim to explore the role of social health in cognitive decline and the onset of dementia. Social health has been defined by the World Health Organization in 1946 as the social domain of health, alongside mental and physical health (7). Our hypothesis is that social health is a driver for the use of cognitive reserve through active facilitation and utilization of social resources of individuals and that poor social health has a substantial influence on cognitive decline and the onset of dementia.

Associations between social characteristics or markers and cognitive decline and dementia are well-documented (8–18). Markers of poor social health, such as poor social engagement and social isolation, are associated with incident dementia (9–12, 18, 19). Although associations of loneliness with dementia risk remain inconsistent (11, 12), social markers such as frequency of contacts, social engagement, social network and (social) leisure activities are associated with better cognitive functioning which may contribute to cognitive reserve and maintenance of cognitive function in old age (10, 14, 18, 20).

Since social health is an umbrella concept, combinations of social health markers might provide added value to knowledge of the development of dementia. Several studies show that a combination of social health markers including social isolation, living alone, inability to help (feelings of not being able to help others), limited social participation and not talking to others every day, is associated with both the development of dementia and reduced cognitive functioning (21–23). A combination of social health markers including participation in social, mental and physical leisure activities and demographic factors such as having high occupational complexity appear protective against cognitive decline (24, 25). Interestingly, a broad spectrum of leisure activities may also be more beneficial than engagement in just one activity (26). These protective “lifestyle factors” are also seen in studies of the development of dementia (27, 28). The combination of four social health markers and one demographic marker is associated with a lower likelihood of developing incident dementia e.g., being married, having support from family members, having contact with friends, participating in community groups and the demographic marker of engaging in paid work (29). Combined scores were linearly associated with incident dementia where those with the highest scores were 46% less likely to develop dementia compared with those in the lowest category (29).

The current associations only address a limited scope of potentially relevant social health markers, underscoring the lack of clarity of the concept of social health. Social health requires a clear definition to advance research into its associations and mechanisms. Currently, many terms are used interchangeably, including social network (30), social integration (30), social engagement (31), social functioning (32), social capital (33), and social contact (18).

Conceptual advancement is needed. This requires a strategic concept–driven effort, that incrementally builds from a conceptual meaning to a more precisely defined unit of meaning (34). In that way we develop a conceptual framework which provides a system to organize thinking about this complex phenomenon (35).

A conceptual framework with an overarching definition of social health, that covers the spectrum of social health domains and potential markers can advance the definition of social health to facilitate the identification of modifiable risk and protective factors, which may in turn shape new avenues for preventive interventions.

Our aim is to develop a conceptual framework for social health to aid conceptual clarity and more comprehensive assessment of social health in future studies.

## Development of conceptual framework

### Study context

The INTERDEM network (pan-European multidisciplinary network of dementia researchers)^1^ profiled social health in the context of dementia (36–40) and initiated the “Social Health And Reserve in the Dementia patient journey (SHARED)” project, funded by the European Union’s Joint Program Neurodegenerative Diseases (JPND 733051082)^2^. SHARED involves several studies with the aim to unravel the interplay between social health and biological and psychological factors. Studies include the development of a conceptual framework for social health, epidemiological studies (41, 42), a systematic review on factors influencing cognitive health (17), a Group Model Building (GMB) study (43), a current study to identify social health measures and an ongoing qualitative study. The conceptual framework was iteratively developed by the interdisciplinary Social Health Stream of SHARED, consisting of a team of 11 experts, and discussed with the whole consortium. The consortium consisted of experts from The Netherlands (*n* = 5), Poland (*n* = 5), Germany (*n* = 4), Australia (*n* = 2), UK (*n* = 1), and Italy (*n* = 1), that were from the disciplines of: psychology (*n* = 5), public health/health services and nursing research (*n* = 4), medicine: geriatrics, psychiatry, neuropsychiatry, and family practice (*n* = 4), epidemiology (*n* = 3), and sociology (*n* = 2).

### Process

Our effort to profile social health started with the conceptualization of Huber et al. (44) focusing on the active role of the individual. This was applied to dementia (36, 39) and to the hypothesis of the SHARED research proposal. In response to questions about the individual focus, we explored underpinnings of the conceptual framework using the following steps: underpinning for concept advancement; concept advancement; and exploration of feasibility of the conceptual framework. Concept advancement used a synthesis of the underpinning studies and an iterative process in which final consensus on the conceptual framework was achieved at the consortium.

### Underpinning for concept advancement

We synthesize theoretical models, conceptualizations, and epidemiological evidence.

The theoretical models that constitute our knowledge of how associations between social health and cognitive functioning and dementia might work through social activity and brain mechanisms are the Social Network Model (31) and the Cognitive Reserve Hypothesis (45). The Social Network Model rests on the assumption that the social structure of the network itself shapes the flow of resources which determines access to opportunities and constraints on behavior (30). It suggests pathways by which networks might influence health status, but does not include cognitive health status as an outcome (30). Additional to social engagement of the individual, this model focuses on the specific characteristics and functioning of the social environment.

The Cognitive Reserve Hypothesis suggests that the brain actively attempts to cope with age-related pathological changes by using preexisting neurobiological capital (e.g., neurons/synapses) to enhance brain network efficiency and flexibility, thus enabling the individual to cope with neuropathological changes (46). This results in maintenance of the level of cognitive function, thus postponing the clinical manifestation of dementia (47). Cognitive reserve reflects the adaptability of the brain in maintaining cognitive abilities or day-to-day function, despite brain aging, pathology, or injury (46, 48). Fundamentally the cognitive reserve concept is one of effect modification: for a given level of neuropathology, this is less clinically expressed (including, but not limited to, cognitive function) in those with higher “reserve” (49). Previous studies have used different approaches to measure cognitive reserve. The most common approach is proxy-based, where education (years or attained level) has been the most commonly studied (46). Other proxies included psychosocial and lifestyle factors such as occupational complexity and engaging in physical and cognitive activities (50).

For the conceptualization, we build on the basic sociological description of the term “social” (51), the WHO definition of health as “a state of complete physical, mental and social wellbeing” (7) and the response to the WHO definition by Huber et al. (44) stating that health might not be a state of complete wellbeing, but the ability to adapt to health challenges and to self-manage. This is especially but not exclusively relevant for advancing age (44).

The key sociological definition refers to social phenomena as a consequence of human interactions, and the influence of one person on another (51). Huber et al. (44) define social health as the influence of social conditions in achieving a balance between capacities and limitations.

Epidemiological evidence reveals markers that refer to the social environment and to the participation of the individual in social life (8–17).

From the social network model, the conceptualizations and the epidemiological evidence we learn that both the individual and the social environment have an active role. Therefore, our conceptualization includes both individual and social environmental levels.

Social health is essentially a relational concept in which wellbeing is defined on the one hand, as the impact that an individual has on others (social environment), and on the other hand as the impact that the social environment (others) has, in turn, on the individual.

These underpinnings have consequences for our past work on social health and we therefore refine our hypothesis as follows: Social health acts as a driver for stimulating the development and the use of cognitive reserve through active facilitation and utilization of the individual’s capacities and those of their social environment. This slows cognitive impairment or maintains cognitive functioning in old age.

### Concept advancement: A conceptual framework for social health

The underpinnings outlined provide us with a concept that is built on theoretical, conceptual and empirical work. Yet, conceptual advancement is needed since the concept remains abstract and hard to operationalize. Therefore, we developed a conceptual framework where meaning is elaborated into more precisely defined domains. The first step is the distinction between the levels of the individual and the social environment. Second, to further facilitate operationalization, we also distinguish domains at both levels. This now allows use of the conceptual framework to identify social health markers and instruments to measure these markers.

#### Individual level

The individual level represents the competences of the individual to act in social life. For the elaboration on this level, we use the domain-related definition of Huber et al. (44). They describe social health as a dynamic balance between capacities and limitations, determined by the individual’s ability to adapt and self-manage to (social) challenges (44). They distinguish the following domains:

- The capacity to fulfill one’s potential (according to one’s abilities and talents) and obligations (i.e., social demands). This refers to compliance with social norms (38). Epidemiological studies related to cognitive functioning and dementia did not identify any markers reflecting this domain (8–18).

-The ability to manage life with some degree of independence (i.e., preserving autonomy and solving problems in daily life). Autonomy refers to acting according to one’s own free will, following own norms and habits to some degree (52). Epidemiological studies related to cognitive functioning and dementia did not identify any markers reflecting this domain (8–18).

- The ability to actively participate in social activities refers to joining activities with others and there is both theoretical and epidemiological evidence for markers in this domain (4, 8, 18).

#### Social environmental level

The immediate social environment has structural and functional domains that are well-established in social network research. As shown below, we added the domain of appraisal of the quality of relationships, as our consortium discussions concluded that appraisal can differ from what can be actually observed. We distinguish the following domains:

-Structure refers to the social ties between persons in networks (e.g., social network size and composition) (14).

-Function refers to actual exchanges between network members, e.g., emotional support and instrumental aid (30, 53). A marker identified in epidemiological research is having support from family members (29).

-Appraisal of the quality of the relationship and interaction refers to perceptions and interpretations. These might differ from actual interactions. An example of its markers is loneliness (10). Social contacts can be perceived as positive or negative, and can be challenging (54).

**FIGURE 1:**
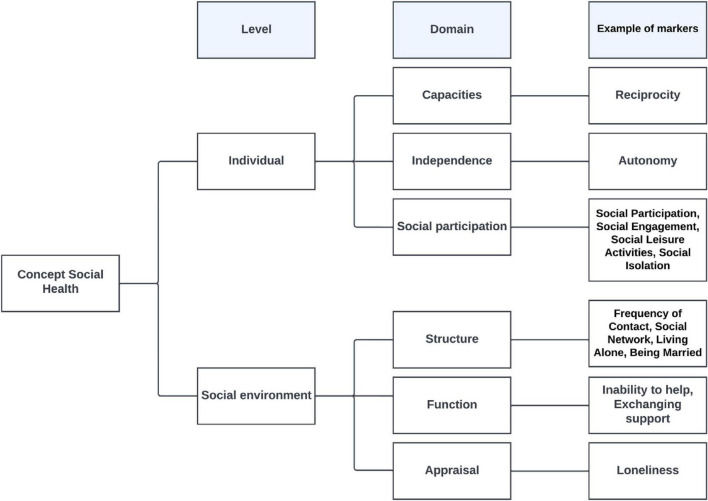
Conceptual framework for social health.

Important novelty value of this conceptual framework is its emphasis on the fact that in the case of dementia an individual’s functioning depends not only on their own capacities. The behavior of their social environment which may support but also hinder them using their capacities may be equally important. This effort now provides a framework for identifying social health markers. Future research could study the inter-relationships within the framework. See Figure 1 Conceptual framework for social health.

## Exploration of potential feasibility of the conceptual framework

The conceptual framework was developed as an aid to identify social health markers in the SHARED project. But does it work like that? We explore the feasibility, meaning the applicability, of the conceptual framework through its use in the following studies of the SHARED project: the review of epidemiological studies (17), the epidemiological associations between social health and cognitive functioning in the SHARED data bases (41, 42) and a Group Model Building (GMB) (43).

The review of reviews includes 314 studies with social factors constituting 10% of all identified factors (17). The epidemiological studies use data bases of five (41) and 13 longitudinal cohort studies (42). These studies are scrutinized to identify social health markers using the conceptual framework. At the individual level, markers are only identified for the domain of social participation, but at the level of the social environment social health markers are identified for all domains.

Since epidemiological research only provides information on what is available in databases, we applied other methodologies to get information on the broad spectrum of social health markers.

In the Group Model Building (GMB) study, a participatory method for involving experts in developing models, we used the systematic review and SHARED’s experts consensus (55). The new GMB information included markers at the level of the individual: the capacity to fulfill norms and obligations (e.g., reciprocity) and independence (e.g., autonomy) (43). These two GMB markers are added to the conceptual framework as domain examples, the other domain marker examples are derived from epidemiological studies. See Figure 1 Conceptual framework for social health.

The conceptual framework is currently being used to classify measures (56) and in the ongoing qualitative study to probe additional relevant social markers.

In sum, the conceptual framework facilitated identification of domain related markers in the SHARED project, thus showing its potential feasibility. Without this framework it would have been hard to systematically identify social health markers or gaps in social health data in current epidemiological research.

## Discussion

We developed a conceptual framework for social health and fine-tuned our hypothesis. The synthesis of theoretical, conceptual and epidemiological resources, led to the recognition of social health relying on the impact of capacities of both the individual and the social environment. This is reflected in the conceptual framework with domains at the individual level referring to aspects of functioning of the individual in social life e.g., being able to fulfill social obligations, while preserving a certain amount of independence and actual participation in social life. The domains at the social environmental level refer to its structure, to social ties that are in place; its functions (what people do), and to the appraisal of the social environment (perception of the quality of social life). These domains resonate with recent work which outlines three similar social environmental life course domains (57).

Our hypothesis linking neuropsychiatric and social sciences extends thinking beyond a predominantly biological concept and touches upon the complexity of dementia etiology.

The proof of the pudding is in the eating. Feasibility exploration demonstrates proof of concept through practical use of the framework in identification of social health markers in the SHARED project’s studies. Our exploration broadened the scope of social heath in the context of cognitive decline from focus on the capacities of the individual (44) to a concept including the capacities of the social environment. Our work expands the 2020 Lancet Commission Report (18), by providing a conceptual framework for identification of social health markers, and thereby potential social risk and protective factors. The conceptual framework remains flexible for novel ideas that can fit under the umbrella concept. Thus, there is scope for the organic growth of new knowledge in this area.

New knowledge is required regarding the interrelationship between the constituent domains at each level and between the individual and the social environmental levels. For now, social health acts as the sum of constituent domains. However, there may be compensation within and between the levels. At the individual level balancing between adaptation to norms, self-management and independency are aspects that require study. We hypothesize that at the environmental level the structural and functional domains are interrelated because they depend on each other. The social structure provides the base for social interaction and social functioning maintains the social structure. Appraisal, personal perception, provides the linking pin between the two levels. The perception of the quality of social environment by the individual offers a key target for intervention research into the interplay between cognitive and social health.

### Interventions

The ultimate goal is to address modifiable social health risks and protective factors in preventive interventions. Interventions can take place at a clinical level and should adopt an individually tailored approach (45), since “one size does not fit all.’ Interventions at a public health level can raise awareness of the importance of social participation for brain health. The results of the first large dementia prevention trial showing a benefit on cognition, the Finnish Geriatric Intervention Study to Prevent Cognitive Impairment and Disability (FINGER), indicated that multi-domain lifestyle-based intervention including diet, exercise, blood pressure monitoring and stimulation of social interaction through activities could contribute to the prevention of cognitive and functional decline amongst at-risk older people (58, 59).

### Delineations

Our conceptual framework describes what constitutes social health; it does not suggest pathways to social health. We see social health from the perspective of the immediate social circle. We consider socioeconomic and cultural characteristics as factors influencing social health. Likewise personal characteristics (e.g., introversion) are considered as internal psychological factors that influence social health but are not a constituent part of social health. While our focus is on the onset of dementia, we acknowledge the important role of social health in living with dementia (60).

### Limitations

The epidemiological studies included in the development of our conceptual framework used associations, so causality cannot be imputed. The study of possible causal mechanisms will be a major challenge. The databases of the epidemiological studies do not include the full spectrum of social health domains, as only social participation is noted at the individual level. Additionally, studies do not always use validated measures (e.g., for loneliness). A broader spectrum of social health markers should be included in epidemiological databases and valid tools for measuring domain-related aspects are needed. Another limitation is that this conceptual framework is a based on western studies. A more diverse perspective is needed for future work (61).

## Conclusion

This conceptual framework has scope for identifying and structuring social health markers relevant in the context of cognitive decline and dementia. Our SHARED project demonstrates its potential feasibility and highlights gaps for relevant future epidemiological research. This is an important first step toward the identification of modifiable risks and factors, which may in turn shape new avenues for preventive interventions.

We therefore suggest that social health is recognized and included in future (epidemiological) studies as one of the factors influencing cognitive functioning and dementia. It is now time to highlight the paradigm of social health in dementia as a priority for dementia research.

## Author contributions

MV-D, MP, and KW-O designed the study. MV-D coordinated the study and drafted the manuscript. SS, ML-B, DS, A-KW, JM, IV, HW, and AM applied the conceptual framework. All authors contributed to the manuscript and agreed with the submitted version.

## Footnotes

1
www.interdem.org

2
https://www.shared-dementia.eu/

## References

[1] Nichols E Steinmetz J Vollset S Fukutaki K Chalek J Abd-Allah F Estimation of the global prevalence of dementia in 2019 and forecasted prevalence in 2050: an analysis for the global burden of disease study 2019. *Lancet Public Health.* (2022) 7:e105–25. 10.1016/S2468-2667(21)00249-8 34998485 PMC8810394

[2] Hardy J Selkoe D. The amyloid hypothesis of Alzheimer’s disease: progress and problems on the road to therapeutics. *Science.* (2002) 297 353–6. 12130773 10.1126/science.1072994

[3] Richard E den Brok M van Gool W. Bayes analysis supports null hypothesis of anti-amyloid beta therapy in Alzheimer’s disease. *Alzheimer’s Dementia.* (2021) 17:1051–5. 10.1002/alz.12379 34057297 PMC8251763

[4] Marseglia A Darin-Mattsson A Kalpouzos G Grande G Fratiglioni L Dekhtyar S Can active life mitigate the impact of diabetes on dementia and brain aging? *Alzheimer’s Dementia.* (2020) 16:1534–43. 10.1002/alz.12142 32715606

[5] Wolters F Chibnik L Waziry R Anderson R Berr C Beiser A Twenty-seven-year time trends in dementia incidence in Europe and the United States: the alzheimer cohorts consortium. *Neurology.* (2020) 95:e519–31. 10.1212/WNL.0000000000010022 32611641 PMC7455342

[6] Winblad B Amouyel P Andrieu S Ballard C Brayne C Brodaty H Defeating Alzheimer’s disease and other dementias: a priority for European science and society. *Lancet Neurol.* (2016) 15:455–532. 10.1016/S1474-4422(16)00062-4 26987701

[7] World Health Organization [WHO]. *Preamble to the constitution of WHO as adopted by the international health conference.* New York, NY: World Health Organization (1946).

[8] Fratiglioni L Wang H. Brain reserve hypothesis in dementia. *J Alzheimer’s Disease.* (2007) 12:11–22. 10.3233/JAD-2007-12103 17851191

[9] Kuiper J Zuidersma M Oude Voshaar R Zuidema S van den Heuvel E Stolk R Social relationships and risk of dementia: a systematic review and meta-analysis of longitudinal cohort studies. *Ageing Res Rev.* (2015) 22:39–57. 10.1016/j.arr.2015.04.006 25956016

[10] Penninkilampi R Casey A Singh M Brodaty H. The association between social engagement, loneliness, and risk of dementia: a systematic review and meta-analysis. *J Alzheimer’s Dis.* (2018) 66:1619–33. 10.3233/JAD-180439 30452410

[11] Rafnsson S Orrell M d’Orsi E Hogervorst E Steptoe A. Loneliness, social integration, and incident dementia over 6 years: prospective findings from the english longitudinal study of ageing. *J Gerontol B Psychol Sci Soc Sci.* (2020) 75:114–24. 10.1093/geronb/gbx087 28658937 PMC6909434

[12] Sutin A Stephan Y Luchetti M Terracciano A. Loneliness and risk of dementia. *J Gerontol B Psychol Sci Soc Sci.* (2018) 75:1414–22. 10.1093/geronb/gby112 30365023 PMC7424267

[13] Bellou V Belbasis L Tzoulaki I Middleton L Ioannidis J Evangelou E. Systematic evaluation of the associations between environmental risk factors and dementia: an umbrella review of systematic reviews and meta-analyses. *Alzheimer’s Dementia.* (2017) 13:406–18. 10.1016/j.jalz.2016.07.152 27599208

[14] Kelly M Duff H Kelly S McHugh Power J Brennan S Lawlor B The impact of social activities, social networks, social support and social relationships on the cognitive functioning of healthy older adults: a systematic review. *Syst Rev.* (2017) 6:259. 10.1186/s13643-017-0632-2 29258596 PMC5735742

[15] Fratiglioni L Paillard-Borg S Winblad B. An active and socially integrated lifestyle in late life might protect against dementia. *Lancet Neurol.* (2004) 3:343–53. 10.1016/S1474-4422(04)00767-7 15157849

[16] Livingston G Sommerlad A Orgeta V Costafreda S Huntley J Ames D Dementia prevention, intervention, and care. *Lancet.* (2017) 390:2673–734. 10.1016/S0140-6736(17)31363-6 28735855

[17] Lenart-Bugla M Łuc M Pawłowski M Szcześniak D Seifert I Wiegelmann H What do we know about social and non-social factors influencing the pathway from cognitive health to dementia? a systematic review of reviews. *Brain Sci.* (2022) 12:1214. 10.3390/brainsci12091214 36138950 PMC9497077

[18] Livingston G Huntley J Sommerlad A Ames D Ballard C Banerjee S Dementia prevention, intervention, and care: 2020 report of the lancet commission. *Lancet.* (2020) 396:413–46. 10.1016/S0140-6736(20)30367-6 32738937 PMC7392084

[19] Piolatto M Bianchi F Rota M Marengoni A Akbaritabar A Squazzoni F. The effect of social relationships on cognitive decline in older adults: an updated systematic review and meta-analysis of longitudinal cohort studies. *BMC Public Health.* (2022) 22:278. 10.1186/s12889-022-12567-5 35148704 PMC8831686

[20] Hahn C Lee C. A brief review of paradigm shifts in prevention of Alzheimer’s disease: from cognitive reserve to precision medicine. *Front Psychiatry.* (2019) 10:786. 10.3389/fpsyt.2019.00786 31736804 PMC6837073

[21] Tsutsumimoto K Doi T Makizako H Hotta R Nakakubo S Makino K Association of social frailty with both cognitive and physical deficits among older people. *J Am Med Dir Assoc.* (2017) 18:603–7. 10.1016/j.jamda.2017.02.004 28411094

[22] Yang Y Yeung W Feng Q. Social exclusion and cognitive impairment - a triple jeopardy for Chinese rural elderly women. *Health Place.* (2018) 53:117–27. 10.1016/j.healthplace.2018.07.013 30114655

[23] MaLSunFTangZ. Social frailty is associated with physical functioning, cognition, and depression, and predicts mortality. *J Nutr Health Aging.* (2018) 22:989–95. 10.1007/s12603-018-1054-0 30272104

[24] Opdebeeck C Matthews F Wu Y Woods R Brayne C Clare L. Cognitive reserve as a moderator of the negative association between mood and cognition: evidence from a population-representative cohort. *Psychol Med.* (2018) 48:61–71. 10.1017/S003329171700126X 28521844

[25] Zhu X Qiu C Zeng Y Li J. Leisure activities, education, and cognitive impairment in Chinese older adults: a population-based longitudinal study. *Int Psychogeriatr.* (2017) 29:727–39. 10.1017/S1041610216001769 28067190 PMC6643295

[26] Karp A Paillard-Borg S Wang H Silverstein M Winblad B Fratiglioni L. Mental, physical and social components in leisure activities equally contribute to decrease dementia risk. *Dement Geriatr Cogn Disord.* (2006) 21:65–73. 10.1159/000089919 16319455

[27] Almeida-Meza P Steptoe A Cadar D. Markers of cognitive reserve and dementia incidence in the english longitudinal study of ageing. *Br J Psychiatry.* (2021) 218:243–51. 10.1192/bjp.2020.54 32223764 PMC7529639

[28] Wang H MacDonald S Dekhtyar S Fratiglioni L. Association of lifelong exposure to cognitive reserve-enhancing factors with dementia risk: a community-based cohort study. *PLoS Med.* (2017) 14:e1002251. 10.1371/journal.pmed.1002251 28291786 PMC5349652

[29] Saito T Murata C Saito M Takeda T Kondo K. Influence of social relationship domains and their combinations on incident dementia: a prospective cohort study. *J Epidemiol Community Health.* (2018) 72:7–12. 10.1136/jech-2017-209811 29089367 PMC5753026

[30] Berkman L Glass T Brissette I Seeman T. From social integration to health: Durkheim in the new millennium. *Soc Sci Med.* (2000) 51:843–57. 10.1016/s0277-9536(00)00065-4 10972429

[31] Berkman L. Which influences cognitive function: living alone or being alone? *Lancet.* (2000) 355:1291–2. 10.1016/S0140-6736(00)02107-3 10776738

[32] Sommerlad A Singleton D Jones R Banerjee S Livingston G. Development of an instrument to assess social functioning in dementia: the social functioning in dementia scale (SF-DEM). *Alzheimers Dement.* (2017) 7:88–98. 10.1016/j.dadm.2017.02.001 28317009 PMC5344217

[33] Van Deth J. Measuring social capital: orthodoxies and continuing controversies. *Int J Soc Res Methodol.* (2003) 6:79–92. 10.1080/13645570305057

[34] Penrod J Hupcey J. Concept advancement: extending science through concept-driven research. *Res Theory Nurs Pract.* (2005) 19:231–41. 10.1891/rtnp.2005.19.3.231 16144241

[35] Emans R. A schema for classifation of conceptual frameworks involving reading. *J Read Behav.* (1970-71) 3:15–21. 10.1080/10862967009546931

[36] Vernooij-Dassen M Jeon Y. Social health and dementia: the power of human capabilities. *Int Psychogeriatr.* (2016) 28:701–3. 26955802 10.1017/S1041610216000260

[37] Vernooij-Dassen M Moniz-Cook E Jeon Y. Social health in dementia care: harnessing an applied research agenda. *Int Psychogeriatr.* (2018) 30:775–8. 10.1017/S1041610217002769 29970212

[38] Dröes R Chattat R Diaz A Gove D Graff M Murphy K Social health and dementia: a European consensus on the operationalization of the concept and directions for research and practice. *Aging Mental Health.* (2017) 21:4–17. 10.1080/13607863.2016.1254596 27869503

[39] de Vugt M Droes R. Social health in dementia. Towards a positive dementia discourse. *Aging Ment Health.* (2017) 21:1–3. 10.1080/13607863.2016.1262822 28098498

[40] Steyaert J Deckers K Smits C Fox C Thyrian R Jeon Y Putting primary prevention of dementia on everybody’s agenda. *Aging Ment Health.* (2021) 25:1376–80. 10.1080/13607863.2020.1783514 32590910

[41] Maddock JG Wolters F Stafford J Marseglia A Dekhtyar S Lenart-Bugla M Social health and change in cognitive capability among older adults: findings from four European longitudinal studies. *medRxiv.* (2022). [Preprint]. 10.1101/2022.08.29.22279324 37497894

[42] Samtani S Mahalingam G Lam B Lipnicki D Lima-Costa M Blay S Associations between social connections and cognition: a global collaborative individual participant data meta-analysis. *Lancet Healthy Longev.* (2022) 3:e740–53. 10.1016/S2666-7568(22)00199-4 36273484 PMC9750173

[43] Seifert I Wiegelmann H Lenart-Bugla M Łuc M Pawłowski M Rouwette E Mapping the complexity of dementia: factors influencing cognitive function at the onset of dementia. *BMC Geriatr.* (2022) 22:507. 10.1186/s12877-022-02955-2 35725402 PMC9208220

[44] Huber M Knottnerus J Green L van der Horst H Jadad A Kromhout D How should we define health? *BMJ.* (2011) 343:d4163. 10.1136/bmj.d4163 21791490

[45] Lisko I Kulmala J Annetorp M Ngandu T Mangialasche F Kivipelto M. How can dementia and disability be prevented in older adults: where are we today and where are we going? *J Intern Med.* (2020) 289:807–30. 10.1111/joim.13227 33314384 PMC8248434

[46] Stern Y Arenaza-Urquijo E Bartrés-Faz D Belleville S Cantilon M Chetelat G Whitepaper: defining and investigating cognitive reserve, brain reserve, and brain maintenance. *Alzheimers Dement.* (2020) 16:1305–11. 10.1016/j.jalz.2018.07.219 30222945 PMC6417987

[47] Stern Y. Cognitive reserve in ageing and Alzheimer’s disease. *Lancet Neurol.* (2012) 11:1006–12. 10.1016/S1474-4422(12)70191-6 23079557 PMC3507991

[48] Stern Y Barnes C Grady C Jones R Raz N. Brain reserve, cognitive reserve, compensation, and maintenance: operationalization, validity, and mechanisms of cognitive resilience. *Neurobiol Aging.* (2019) 83:124–9. 10.1016/j.neurobiolaging.2019.03.022 31732015 PMC6859943

[49] Richards M Deary IJ. A life course approach to cognitive reserve: a model for cognitive aging and development? *Ann Neurol.* (2005) 58:617–22. 10.1002/ana.20637 16178025

[50] Fratiglioni L Marseglia A Dekhtyar S. Ageing without dementia: can stimulating psychosocial and lifestyle experiences make a difference? *Lancet Neurol.* (2020) 19:533–43. 10.1016/S1474-4422(20)30039-9 32470425

[51] de Jager H. *Grondbeginselen der sociologie (Basic Principles of sociology).* Leiden, NL: Noordhoff Uitgevers (1974).

[52] Vernooij-Dassen M Osse B Schade E Grol R. Patient autonomy problems in palliative care: systematic development and evaluation of a questionnaire. *J Pain Symptom Manage.* (2005) 30:264–70. 10.1016/j.jpainsymman.2005.03.010 16183010

[53] Pescosolido B. *The sociology of social networks.* New York, NY: Sage Publications (2007). 10.4135/9781412939645.n20

[54] Walen HL. Social support and strain from partner, family, and friends: costs and benefits for men and women in adulthood. *J Soc Personal Relationsh.* (2000) 17:5–30. 10.1111/1467-9566.13386 34655081

[55] Rouwette EV Thijssen C. Group model building: a decision room approach. *Simul Gaming.* (2000) 31:359–79. 10.1177/104687810003100303

[56] Wiegelmann H Lenart-Bugla M Vernooij-Dassen M Verspoor E Seifert I Szczesniak D *Measurement of Social Health in the Context of Cognitive Decline and Dementia: Protocol for a Systematic Review.* (2020). 10.17605/OSF.IO/8J9YT PMC11599264 39605998

[57] Fuller H Ajrouch K Antonucci T. The convoy model and later-life family relationships. *J Fam Theory Rev.* (2020) 12:126–46. 10.1111/jftr.12376 32536976 PMC7283809

[58] Kivipelto M Solomon A Ahtiluoto S Ngandu T Lehtisalo J Antikainen R The finnish geriatric intervention study to prevent cognitive impairment and disability (FINGER): study design and progress. *Alzheimer’s Dementia.* (2013) 9:657–65. 10.1016/j.jalz.2012.09.012 23332672

[59] Ngandu T Lehtisalo J Solomon A Levälahti E Ahtiluoto S Antikainen R A 2 year multidomain intervention of diet, exercise, cognitive training, and vascular risk monitoring versus control to prevent cognitive decline in at-risk elderly people (FINGER): a randomised controlled trial. *Lancet.* (2015) 385:2255–63. 10.1016/S0140-6736(15)60461-5 25771249

[60] Samtani S Stevens A Brodaty H. Preserving and enhancing social health in neurocognitive disorders. *Curr Opin Psychiatry.* (2021) 34:157–64. 10.1097/YCO.0000000000000683 33395096

[61] van Wezel N Francke A Kayan-Acun E Ljm Devillé W van Grondelle N Blom M. Family care for immigrants with dementia: the perspectives of female family carers living in the Netherlands. *Dementia.* (2016) 15:69–84. 10.1177/1471301213517703 24403313

